# Multiple vehicle cooperation and collision avoidance in automated vehicles: survey and an AI-enabled conceptual framework

**DOI:** 10.1038/s41598-022-27026-9

**Published:** 2023-01-12

**Authors:** Abu Jafar Md Muzahid, Syafiq Fauzi Kamarulzaman, Md Arafatur Rahman, Saydul Akbar Murad, Md Abdus Samad Kamal, Ali H Alenezi

**Affiliations:** 1 Faculty of Computing, Universiti Malaysia Pahang, Pahang, 26600 Malaysia; 2 School of Engineering, Computing and Mathematical Sciences, University of Wolverhampton, Birmingham, WV1 1LY UK; 3 Graduate School of Science and Technology, Gunma University, Kiryu, 376-8515 Japan; 4 Electrical Engineering Department, Northern Border University, P.O.Box: 1321, Arar, Saudi Arabia

**Keywords:** Computational science, Computer science

## Abstract

Prospective customers are becoming more concerned about safety and comfort as the automobile industry swings toward automated vehicles (AVs). A comprehensive evaluation of recent AVs collision data indicates that modern automated driving systems are prone to rear-end collisions, usually leading to multiple-vehicle collisions. Moreover, most investigations into severe traffic conditions are confined to single-vehicle collisions. This work reviewed diverse techniques of existing literature to provide planning procedures for multiple vehicle cooperation and collision avoidance (MVCCA) strategies in AVs while also considering their performance and social impact viewpoints. Firstly, we investigate and tabulate the existing MVCCA techniques associated with single-vehicle collision avoidance perspectives. Then, current achievements are extensively evaluated, challenges and flows are identified, and remedies are intelligently formed to exploit a taxonomy. This paper also aims to give readers an AI-enabled conceptual framework and a decision-making model with a concrete structure of the training network settings to bridge the gaps between current investigations. These findings are intended to shed insight into the benefits of the greater efficiency of AVs set-up for academics and policymakers. Lastly, the open research issues discussed in this survey will pave the way for the actual implementation of driverless automated traffic systems.

Over the last decade, the scientific community has been paying close attention to research into sustainable technologies, artificial intelligence and smart city. This trend will continue in the coming years^1^. One area that has undergone intensive investigation is the public transportation service, while the automotive industry is heading towards automated vehicles (AVs) intending to boost road safety. It is estimated that 94% of road accidents occur where drivers are primarily responsible due to a lack of proper attention. Whether due to poor visibility or excessive speed, they endanger themselves and others on the road^2^. In this, autonomous vehicles have emerged as a potentially big change that has the potential to eradicate the errors that drivers make while operating their vehicles^3^. Recent AVs collisions during testing, on the other hand, highlight the need for more rigorous risk analysis. In May 2011, the United Nations (UN) launched a global schema titled “Decade of Action for Road Safety 2011–2020”^4^ in response to the high death toll associated with unsafe roads. Modern scientists want to transfer all driving tasks from humans to machines since the majority of traffic collisions (94%) are caused by driver distractions.

The term *multiple collision* refers to a collision that involves two or more vehicles(up to *n*) colliding with one another in the same collision. Altogether, these multiple collisions accounted for almost 20% of all traffic collisions and 18% of the deaths on United States motorways^5^. Furthermore, multiple collisions are responsible for up to 50% of urban traffic congestion^6^. Because of highway conditions, rear-end crashes accounted for 42.7%of all accidents that usually lead to multiple vehicle collisions (MVCs)^7^. Through an extensive evaluation of recent AVs crash data, we found a crucial indication that the AVs systems are most prone to rear-end collisions, the leading cause of chain crashes or crashes among multiple vehicles^8^. Additionally, as the transportation community moves from an era of data-scarce to a generation of data-rich, a standard methodological shift from physics-based methods to artificial intelligence techniques is urgently needed to forecast the transportation dynamics of vehicles operating adjacent to human-driven vehicles and help socially optimize policymakers^9^.

Research on MVCs in AVs highlights the need to follow the evaluation of the consequences of a collision^10^. In contrast, existing research is dedicated to three viewpoints: (1) identifying multiple collisions^11^, (2) analyzing multiple collisions’ characteristics^12^, and (3) multiple collisions’ risk modelling^13^. Collision avoidance at high volume vehicle velocity, which leads to MVCs, is considered a high non-linearity vehicle force that demands an optimal motion planning strategy. The current control strategies are validated only at low and medium velocity; a reliable, validated strategy is essential for high-speed situations^14^. Regrettably, the continuous AVs research focused solely on collision avoidance strategies for two consecutive vehicles and ignored the MVCs aspects. Several review articles and journals which focused on the aspects mentioned above were discussed and compared. Table 1 presents the result of the discussion and comparison.

**Table 1:** Comparison of autonomous vehicle collision (single and multiple vehicles) avoidance related survey papers.

Survey coverage										
Refs.	SP	CC	TA	DM	VC	DS	CI	SVC	MVC	CS
^15^	$\checkmark$	$\checkmark$	$\checkmark$	$\checkmark$	$\checkmark$		$\checkmark$			
^16^	$\checkmark$	$\checkmark$		$\checkmark$	$\checkmark$	$\checkmark$	$\checkmark$			
^17^	$\checkmark$	$\checkmark$	$\checkmark$	$\checkmark$	$\checkmark$	$\checkmark$	$\checkmark$			
^18^	$\checkmark$	$\checkmark$	$\checkmark$	$\checkmark$	$\checkmark$	$\checkmark$				
^19^	$\checkmark$	$\checkmark$		$\checkmark$	$\checkmark$		$\checkmark$	$\checkmark$		
^20^	$\checkmark$	$\checkmark$	$\checkmark$	$\checkmark$	$\checkmark$		$\checkmark$			
Ours	$\checkmark$	$\checkmark$	$\checkmark$	$\checkmark$	$\checkmark$	$\checkmark$	$\checkmark$	$\checkmark$	$\checkmark$	$\checkmark$

*SP* Sensing & perception, *CC* Communication & cooperation, *TA* Threat assessment, *DM* Decision-making, * VC* Vehicle control, *DS* Database & software, *CI* Challenges and issues, *SVCA* Single vehicle collision avoidance, *MVCA* Multiple vehicle collision avoidance, *CS* Conceptual solution.

**Figure 1:**
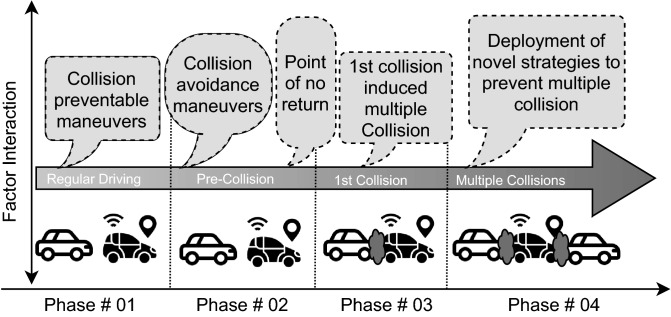
Multiple vehicle collisions illustration in four phases.

However, more intensive research is essential for highlighting principles of examining accidents and preventing chain collisions, which represent the generating mechanism of a traffic accident^21,22^. In support of this argument, a safety framework for driving actions should be built from the viewpoint of a chain collision. The combined potential concerns of MVCs are illustrated by Fig. 1 in four phases: the first phase is the regular driving representation; the second phase is the pre-crash situation associated with the point of no return; the third phase represents the first crash situation; and finally, the fourth phase is the illustration of MVCs induced by the first collision.

Given these challenges, a growing number of researchers are devoted to perfecting the driving strategy of autonomous vehicles (AVs) in order to create reliable ways of avoiding collisions. Real-time vehicle control and planning for smooth driving with enhanced awareness; routing based on microscale traffic data; coordinated platooning in response to traffic signals; these are just some of the features identified in a survey of AVs control and planning architectures^23^. In addition to the potential for AVs to increase safety by mitigating traffic accidents and reducing the traffic crash severity, a recent large-scale study^24^ demonstrated how to combine and merge highway on-ramps into a standard intersection strategy. A comprehensive analysis^25^ can provide us with high-level solutions to improve the ability of autonomous vehicles (AVs) to control themselves in an urban traffic environment by estimating the traffic flow and optimizing signal timing. Apart from that, an extensive discussion was held, focusing on the distributed control mechanisms depending on the dynamical modeling of AVs^26^. Contemporary motion control focuses on the cooperative longitudinal motion of multiple vehicles and is extensively discussed^27^.

During a combined approach, different strategies were suggested that focus either on improving certain areas or considering all difficulties. As a result, in order to gain a thorough understanding of the research progress in this field, it is necessary to compile all available works. Therefore, a comprehensive taxonomy is demonstrated in this article that differentiates techniques, methods, and technology offered to date for effective autonomous driving strategies for single-vehicle collision avoidance (SVCA) and multiple-vehicle collision avoidance (MVCA). Subsequently, we reviewed relevant literature to highlight the key ideas of each current study. Essentially, the purpose of this study is to inspire readers to recognize current research breakthroughs in this domain and identify unsolved concerns. Finally, we offer a conceptual framework of an MVCCA strategy for creating an optimal solution in an AVs system.

The contributing contents of this paper are as follows:

1. A comprehensive analysis that identifies and segments the chain events of collisions associated with MVCs was conducted. Both SVCA and MVCA perspectives are reviewed objectively, and a taxonomy is created that consolidates all potential collision avoidance approaches into a single window.
2. Recent technologies and protocols are investigated to determine realistic automated driving decisions and optimal cooperative decision-making methods. According to their performance matrix, the practical difficulties and issues are presented in depth.
3. This study offers a future research direction with an AI-enabled conceptual framework for MVCCA in AVs. The proposed framework closely scrutinizes five aspects of AVs to guarantee adequate driving strategies. Learning-based monitoring and preservation with highlighted applications are also offered in the intended framework to unlock the potential of AVs as standalone MVCCA strategies.
4. An extensive review of the existing challenges, including the design issues of optimum decision-making and technical matters regarding essential performance aspects of collision avoidance among multiple vehicles. In this context, we proposed a deep reinforcement learning-based decision-making model to control multiple vehicles in a multi-agent traffic environment to perform the best action-state map for our automated agents. The proposed model will work to reform the computational aspects of collision avoidance optimization according to our proposed framework.
5. Finally, open research issues are sketched out to allow future research direction on existing work and potential research domains.

As the paper highlights a comprehensive overview of specific topics relevant to the development of the conceptual framework, it enables readers to uncover these topics. The rest of the paper is laid out as follows, Sect. 2 presents an overview of the AVs and collision segmentation. In Sect. 3, the challenges and issues of avoidance MVCs in AVs are extensively illustrated. Section 4 represents a taxonomy of MVCCA and under this taxonomy in Sect. 5, a spacious AI-enabled conceptual framework is deployed for MVCCA in AVs. Section 6 synchronizes valuable future research indications to provide respected researchers with future challenges. Finally, Sect. 7 concludes the paper by revealing the article’s contribution.

## Overview of MVCCA

**Figure 2:**
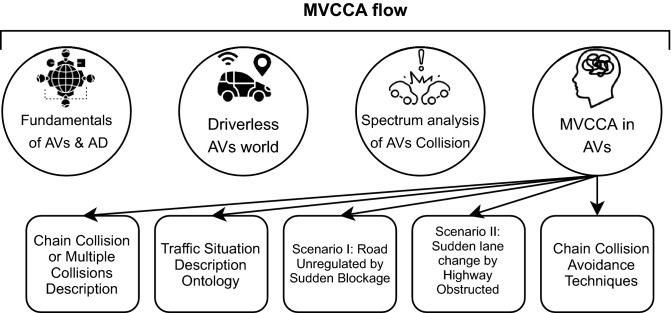
The overview of work flow of MVCCA in AVs.

The leading subject in automotive science in recent years has been AVs^28,29^. Millions of lives are likely to be saved soon, considering the remarkable statistics showing that the number of casualties in road accidents has been reduced to a good 1.2 million a year in the last ten years^30^. Furthermore, it will optimize traffic and reduce travel times significantly. An overview of the workflow is presented in Fig. 2 for the sequences involving multiple vehicles cooperating and avoiding chain collisions.

It is worth mentioning that the strength gain is self-evident in developing a stable AVs system. However, their implementation is a major challenge for both rule-based control and data-driven decision-making. Readers are encouraged to refer to^31,32^ for the appropriate analysis of key technologies for assistance systems for collision prevention. Multiple business giants in different countries are currently working on the production of AVs. The *authors* of^33^ briefly described the data on the growth of each country in AVs design and the challenges faced by those countries. AVs production in the initial days entails numerous studies and problems. Real-world AVs are never 100% sure what various things are such as road boundaries, lanes, rules, signals, and so on, in a situation. Instead, it has a certain level of confidence or degree of certainty about all these aspects^34^.

### AVs fundamentals

**Figure 3:**
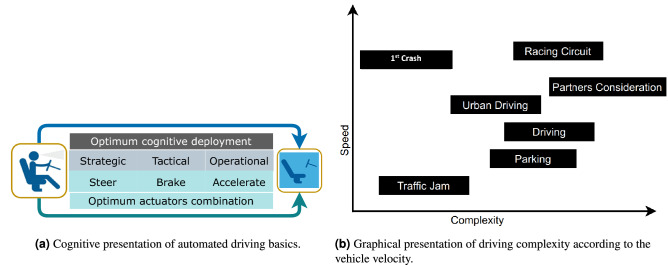
Evaluations of driving complexity in AVs.

In the modern transportation world, autonomous systems have been activated to prevent 94% of driver error road accidents^2^. AVs can sense their surroundings and operate without human interference. Figure 3a is the cognitive presentation of AVs basics. For a more elaborate discussion on the automation level of AVs, readers are referred to^35^. The mass production of tools relevant to AVs is approaching thanks to rapid advancements in AVs technologies, particularly the recent advancements in LiDAR, GPUs, and learning control strategies^33^. Many business giants such as Waymo and GM-affiliated automotive and IT firms are working hard to get their advanced self-driving cars onto public roads as soon as possible. The leading peril of optimal performance in AVs technologies is traffic collisions. Thus, the mechanism for accident prevention must be capable of controlling all types of threats during automated navigation, with the progress of the production of AVs. Figure 3b depicts the complexity and speed of numerous driving conditions.

### *n*-number AVs

**Figure 4:**
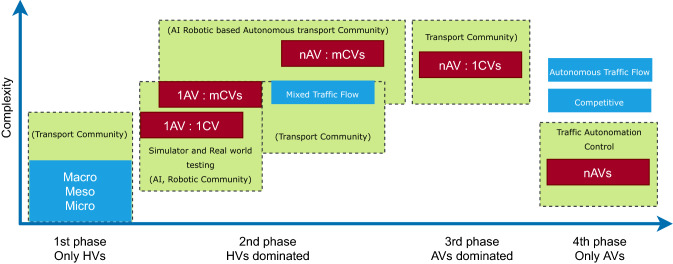
Time with complexity refinement at each phase (according to both researcher and manufacture aspect)^36^.

To date, most of the current research has perhaps concentrated unexpectedly on two polar scenarios, in which either one AV is traveling on a highway in an environment dense with human drivers or an AV network with minimal interaction with human-operated participants. The much more realistic but challenging transformation between these two scenarios has received much less attention. It is this hybrid human-machinery space, now known as *mixed autonomy*, which merits our collective interest. In the survey^36^, authors divided this transformation into 4 stages, pure conventional vehicles (CVs), the CVs-dominated stage, the AVs-dominated stage, and the pure AVs stage. The latter 3 stages are the subject of this article. The difficulty of modelling for each step is shown in Fig. 4. The CVs-dominated and AVs-dominated stages, that is, *mixed autonomy*, are difficult to chart. An understanding of the unknown road participant and complicated interactions among different vehicle types is required. The following concepts further split the relative proportion of AVs and CVs into mixed autonomy (indicated in road map in Fig. 4):

1. *1st stage:* 1 AV + 1 CV (one AV has one CV interaction).
2. *2nd stage:* n AVs + m CVs (Multiple AVs travel a CVs-dominated traffic environment).
3. *3rd stage:* m AVs + n CVs (multiple AVs interact with n CVs in the AVs-dominated traffic environment).
4. *4th stage:* n AVs (a domain for pure AVs) with replacing all conventional vehicles with AVs. (AVs communicate with each other accordingly).

In Fig. 4, a black-dotted box is included in the group associated with each step. The objective of the transport community is pure CVs, then a model which is dominated by CVs, and finally pure AVs dominated^36^. However, researchers are more concerned with the step of the CVs-dominated operation, in which one or few AVs navigate the traffic environment.

### Spectrum analysis of collision in AVs

Most of the current automated driving research goes far beyond the control of a single vehicle. However, in reality, decision-making in crucial scenarios and the initiation of strong sensors, cooperative communications networks, and embedded systems have created extensive concern about how to solve the problem of multiple automated vehicles’ cooperative control. The problems of vehicle control by motion planning for a single automated vehicle are usually divided into three segments: (1) the stabilization of points, (2) tracking trajectory, and (3) the path following^37^. For multiple vehicles, formulating a cooperative trajectory generation strategy is the main issue. In particular, a collision-free route is adopted by each vehicle, and all vehicles reach their respective destinations. In Table 2 is shown the major aspects of single and multiple vehicle collisions^38^.

**Table 2:** Major aspects of SVC and MVC.

Features aspects		SVC	MVC
Crash severity		Less severe than MVC	More severe than SVC
Collision types	Static	Very common	Very common
	Dynamic		
	Uncertain		
Cause pattern		Defined situations	Unpredictable situations
Automated approach		Very common	Very common
Avoidance techniques		Study widely	Study marginally
Avoidance algorithms		Study widely	Study marginally
Single and multi agents perception	Agents	Single	Multi agents
	Environment	Less complicated	Very complicated
	Private goal	Focused	Focused
	Common goal	Not focused	Focused
Cooperation driving	Characteristics of cooperation	Location	Agents involved
		Interaction	Location
		Duration	Urgency and costs
		Preparation time	Interaction type
		Initiation	Duration
			Mutuality
			Preparation time
			Initiation
	Examples of cooperative situations	Lane merge	Platooning
		Truck overtaking	Lane merge
			Truck overtaking

### MVCCA in AVs

As with the conventional traffic system, the autonomous traffic system offers a potential perspective on both the SVC and the MVCs. Despite the fact that automakers have focused on creating realistic solutions for AVs to replace human-driven vehicles, the most recent solutions are only suitable for single vehicles. On the other hand, road traffic is a dynamic and interactive system. Such a system necessitates a multifaceted approach to solving the issue since it takes into account not only the pedestrians and the surrounding road but also other road users, which may involve multiple participants^39^. Authors^40^ extensively investigated and illustrated a region map of single, double, triple, and multiple vehicle collision conditions regarding sudden slowdown. The article^36^ evaluates the steering stability for multiple vehicles in the case of automatic or manual driving, which is restricted for safety. In fact, MVCs are likely to result from a series of unstable coupled groups of vehicles.

#### Chain collision or MVCs description

MVCs are the ultimate result of an SVC in a traffic system. Drivers on highways frequently rely heavily on the vehicle’s tail brake lights to decide if they brake^40^. This creates potentially dangerous situations when a vehicle follows another closely, particularly when the ability to see past the vehicle is limited. The reaction time of the driver between the occurrence and the frequency of the brake is usually 0.75 to 1.5 seconds^41^. There may be few margins of protection if a short inter-vehicle distance is maintained in order to prevent accidents during abrupt braking. Furthermore, the successive drivers’ cumulative reaction times in heavy traffic will lead to a number of secondary accidents and create multiple vehicle accident chains^42^.

#### Traffic situation ontology

The perception process of dangerous situations, in a cooperative group of vehicles, is discussed in^43^. In the meantime, it has been accepted that a higher degree of situational understanding is often required in order to provide driver assistance. Vehicles have to understand the situation they are involved in. This is or will become the basis for numerous implementations, including advanced crash detection and mitigation systems. The advantage of knowing the scene would allow for automated driving of more than one vehicle to deal with hazardous situations at high speed in complex inner-city environments or cooperative maneuvers if necessary^44^. Traffic collisions prevent the flow of traffic, block the highway, and cause serious congestion. Sometimes, the blockage causes collisions between vehicles. The accident often causes further collisions and leads to multiple-vehicle collisions.

#### Scenario I: highway unregulated by sudden slow down or blockages

We consider a typical highway scenario in which *n* number of vehicles are traveling in parallel, in front of or behind each other. All vehicles attempt to monitor their own relationship to speed. In this situation, a driver usually relies on the brake light of the car ahead of them to evaluate their own braking action in road emergencies caused by bad weather or misjudgment^45^. In low-visibility situations, the behaviour of the traffic is certainly different from that in natural conditions. With the use of a model of friction-force^46,47^, collision among multiple vehicles was investigated in low visibility situations. However, if the emergency incident is caused by multiple vehicles ahead, then it could be too late to stop the collision by the time the vehicle brakes immediately ahead. In addition, the combined reaction time of drivers across all the vehicles ahead will further escalate the situation. Consequently, a single emergency incident may also lead to injuries in multiple-chain collisions. Another aspect is that the driver deploys brake matching to the taillights of the leading vehicle and the rearrangement of friction force, which strongly depends on the velocity of the vehicle in the traffic situation. Chain collisions can be caused by the first accident^19,20^.

#### Scenario II: sudden lane change by highway obstructed

**Figure 5:**
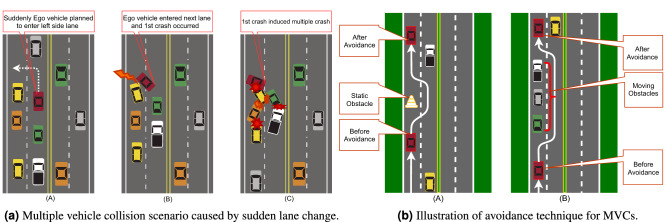
Demonstration of MVCs and its avoidance technique.

The first collision caused by a sudden lane change can induce further collisions and may lead to MVCs among several vehicles when a vehicle switches lanes on a two-lane highway from its ego lane to the next lane. If a vehicle enters the second lane at a high (or low) speed from the first lane, there is a high chance of a first collision with the vehicle that is driving in that lane, and the colliding vehicle could be positioned as the forward (or rear) vehicle, which can cause further collisions to occur as a result of the initial collision^48^. Another scenario is that the three-lane highway is reduced to a single lane due to road work heterogeneity or that the leading vehicle comes to a halt unexpectedly due to a blockage^45^. This roadwork is announced to all vehicles via road signs. According to these signs, vehicles must slow to 70 km/h and then merge into a single lane. As a consequence, the situation becomes more complicated, exposing the differences in the controls. Secondary collisions are typically caused by the first collision, and^49^ contains a thorough investigation of the factors influencing secondary crashes. Potential readers were referred to read^50–52^ to uncover the crucial aspects of secondary collisions. Figure 5a is the symbolic representation of the consequences of MVCs.

#### Chain collision avoidance techniques

While reviewing contemporary research works, we usually found numerous solutions and protocols concerning SVC; however, the MVCs problems continue to suffer from a lack of concern, particularly in the domain of AVs. Traffic situations with multiple vehicles interacting are difficult in AVs system. Even though another traffic participant’s rough intent is understood, all participating vehicles must agree on a cooperative decision that gives a conflict-free trajectory plan, indirectly or explicitly. For each vehicle, the movement must be secure and comfortable and must accommodate all individual goals and desires^53^. The standard interval between these intervals can be determined using the collision-free interval for each agent^54^. When each agent’s velocity is adjusted in parallel, the velocities in the non-intersecting distance inevitably avoid colliding. This agreement protocol is used in competing speeds in a common interval^55^. As a navigation query, collision detection and avoidance in agents^56^ or multi-agent^57–59^ scenarios has also been addressed.

Theoretically, chain collisions can be avoided or decreased in severity by reducing the time between an emergency occurrence and the moment when approaching vehicles are in a chain collision. Propagating a vehicle-to-vehicle incident warning alert is one way to do this. The warning alert is designed to circumvent the usual chain of drivers responding to the activation of vehicle brake lights immediately ahead of them, and even allow drivers to react to an incident before seeing it. Secondary collision mitigation strategies can be found in^11,13^. Common strategies employed for MVCCA include Platooning^60^, Active Brake Control^61^, Time-Critical Cooperative Control^37^, Trajectory Re-planning^62^. Authors in^62^ proposed avoidance technique for chain crash as shown in Fig. 5b.

## Challenges and issues of MVCCA

For combinations of hundred-pulse sensors, communications devices, and actuators, an extensive evaluation will be needed before the mass production of AVs. These matters indicate the analysis of the root causes of AVs failures and finding out the chain events of the potential failures. Obviously, policymakers and researchers are dependent on this kind of comprehensive evaluation to develop the optimum strategies. Several barriers are likely to challenge the advancement and execution of sensible driving technologies, particularly the avoidance of collisions among multiple vehicles. The key factors that could hamper technology adoption before or after its full maturity consist of:

### Mixed traffic systems management

It is proven that technology is not the outcome of one or two days, but rather the outcomes starting from the 1960s (new models) until now. The same is true for transportation systems. We cannot expect all the road transport systems to convert to automated systems within a day^63^. As a result, it is expected that the transition from the shape of a traditional non-automated vehicle fleet to the shape of an AVs fleet would occur in stages over time. This viewpoint implies that our AVs-based framework would take into account both AVs and conventional vehicles (CVs) at the same time. According to the most recent automated vehicle testing findings submitted by AVs testing companies, the majority of AVs involved in accidents are caused by CVs sharing the road with AVs^64^.

### Cooperative maneuvers for each vehicle safety

Research challenges involve expanding the method to random road geometry and incorporating for each vehicle a plan *B* trajectory that ensures that in the case of a crash, e.g., loss of contact, a safe state is reached. Although the measurement of cooperative behavioural action^53,65^ is almost realistic, with a growing number of participants, it does not scale well. In the AVs traffic systems, it is more crucial than it is in conventional traffic systems.

### Multi agent robotics systems

In multi-robot navigation, global path planning and local motion planning play crucial roles. Autonomous driving is clearly a multi-agent, dynamic field, with the most difficult challenge being the deployment of a collision-free, safe, and robust trajectory plan for each of the robots from their starting point to the desired destination. In any unexpected, critical situation, the system needs to be capable of re-planning for a proper collision avoidance strategy. On the other hand, in multi-agent robot environments, where the agent learns the collision avoidance navigation strategies from the environment, it is more challenging to deploy the particular capabilities to find collision-free routes, and they are well adapted to all kinds of unseen scenarios^66^.

### Adequate data for efficient learning

Machine learning algorithms are currently being learned in a supervised method primarily and therefore, adequate data is needed for efficient learning and a robust training process. Despite the fact that automated vehicles have been tested in highly regulated environments, they often struggle to make the right decisions, sometimes with disastrous consequences. For adapting automated navigation to all forms of critical driving environments, defining a deriving mechanism in any certain crucial situation first would benefit the deployment of robust driving ability in all the scenes. The author mentioned some key critical conditions in^67^. Robust schemes, such as re-planning and retreating the perspective process, would be built to accomplish safe and secure planning in the web of uncertainties. Erhan, L. *et al.,* reviewed the anomaly recognition in automated vehicle sensor systems^68^. Table 3 shows the summary of currently available data sets mentioned in prominent survey papers.

**Table 3:** Prominent survey papers represented data-sets with detailed features.

Refs.	Data sets	Factors	Critical scenarios	Challenges to handling
*Survey of the explicate of environmental conditions*				
^69^	ApolloSpace, NightOwls	Illuminati-on	Shadow, directly facing the sun, Night	Light intensity variations.
^70^	AMUSE, CMU, Oxford RobotCar	Weather	Snow, rain, fog	Difficulty in computer vision-based tasks.
^71^	ApolloSpace, Berkeley DeepDrive	Traffic Conditions	High speed, Multiple collisions, Heavy traffic flows.	Lack of realistic datasets
^72^	IDD, CCSAD, Highway work zones	Road conditions	Damage, rough surfaces	Lack of data
*Explicate of behavioral factors*				
^73^	UAH, Argoverse	Vehicle’s behaviors	Lane change, overtaking, high speed	Real-time prediction in multiple participants
^74^	JAAD, Daimler pedestrian	Participants and road users’ behaviors	Crossing, wrong direction movements.	Lack of datasets

### Simulator and simulation studies

Conducting a better-automated system generally requires more and more experiments and reshaping of the systems, and it is not always possible to use a real automated vehicle. In addition, performing more in-depth investigations and configurations that require risky scenarios must be conducted in some type of simulation. Since the 1960s, simulator studies in the automotive domain have been carried out^75^. A simulation does not contain any of the actual driving information, meaning that creating a realistic simulation experience both psychologically and physically remains a challenge. Acquisition of samples, simulator fatigue, training of simulator, interface designing, requests for take-over, and the secondary tasks of automated and simulated driving study are examples of these^76^. Traffic simulator in open-source phase as- SUMO^77^ MATSim^78^ in commercial phase AIMSUN^79^ PTV Vissim^80^ Paramics^81^ VIPS^82^, network simulator 802.11p/ITS G5 protocols^83^ OMNeT++^84^, NS-3^85^, Multi-Agent Systems(MAS) LightJason^86^. Potential readers are invited to read the systematic literature review on Agent-Based Simulation of Autonomous Vehicles^87^. The *authors* of^88^ evaluated the segmented validity checking systems into robustness testing, combination testing, and search-based testing methods. In the field of automated driving, there is a need to bridge the gap between open-source software and vehicle hardware, see^89^ and^86^ for ITS simulation systems, respectively.

**Figure 6:**
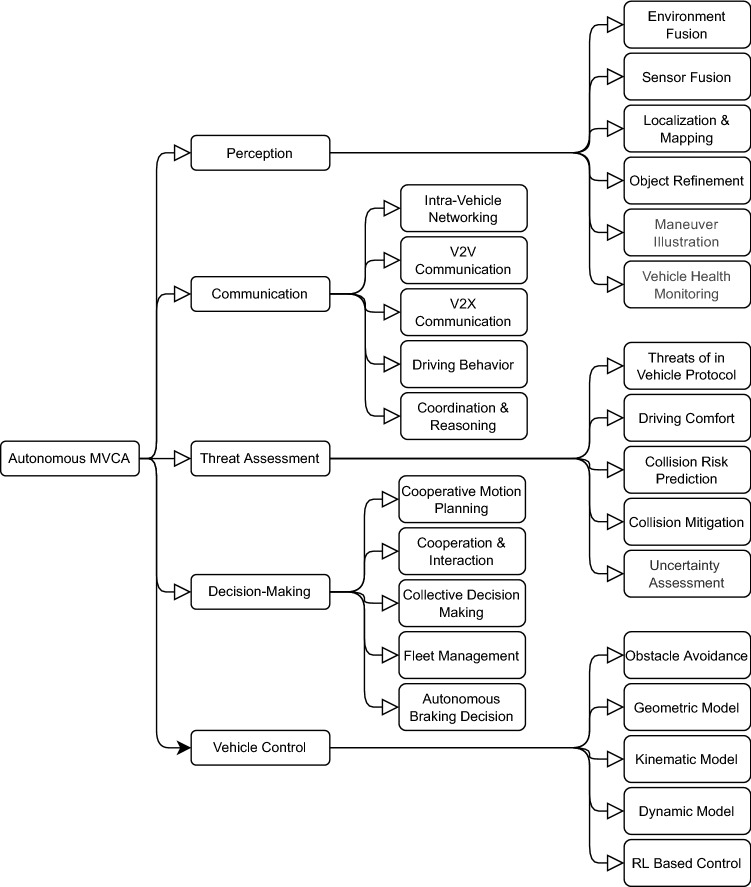
Taxonomy of MVCCA in AVs.

## Taxonomy of MVCCA in AVs

In AVs systems, MVCCA is a more complex task than SVCA, and in this section, according to the existing research publication, we developed an extensive taxonomy. The future of automotive safety is generally predicted to be self-driving and highly AVs, potential academics and manufacturers are conducting crash avoidance and AVs research to keep drivers and passengers safe. The taxonomy is presented by Fig. 6 to address different perspectives and methods for forming MVCCA strategies associated with the SVCA in AVs.

The basics have been categorized based on the literature’s most pressing concerns. To begin, numerous works have been discovered to allow a vehicle to move on its own, with four basic subsystems typically incorporated: location identification and navigation system, environmental situation analysis system, motion planning system, and trajectory control system. The second most prevalent strategy is focused on decision-making model development for vehicle control using physics-based control theories and the latest learning control methods. This section comprises all the collision avoidance technologies proposed by numerous writers. Third, many studies have been found that aim to provide learning control to prevent single-vehicle collisions and adaptive control of single vehicles to optimize the AVs. This segment talks through many forms of perception, communication, threat assessment, decision-making, and vehicle control approach applicable to a distinct range of technologies. Finally, there are a few studies that offer a complete method of MVCCA strategies utilizing a combination of the five fundamental aspects of AVs. Further elaboration of this research will be presented in the following sections.

### Perception

The process of perception is entirely dependent on the domain of sense, and its perfectness is a crucial factor in the AVs system’s collision avoidance strategies. From the perspective of MVCCA, the fundamental problems are the correct understanding of the road traffic environment, the identification of possible traffic accidents, and the proposal of alternative driving strategies. Contemporary object detection and tracking systems such as 3D object detection for automated traffic systems are offered with a multi-modal 360-degree balancing framework proposed by^90^. The perfect perception process is dependent on several facts, and given how crucial this is for MVCCA that we reviewed some articles to determine the focus feature in the perception phase in AVs, and it is shown in Table 4. A more elaborate discussion is found in the following subsections.

#### Environment fusion (EF)

AVs have the power to perform automatic actions and navigate themselves based on their surroundings and pre-programmed duties^91^. Based on the environment in which it is operating, AV systems may have varying levels of complexity. Artificial intelligence (AI) has fueled the improvement and deployment of AVs in the transportation sector. Fuelled by large data from numerous sensing devices and improved computer resources, AI has come to be a vital component of AVs for understanding the surrounding environment and creating appropriate choices in motion. For the ultimate objective of self-driving cars, understanding how AI functions in AV systems is essential^91^.

#### Sensor fusion (SF)

For accurate perception, AVs rely on Sensor Fusion (SF), which requires them to gather input from their surroundings and extract important knowledge in order to classify data by semantic meaning^92^ and even anticipate their future states^91^. To do this, the perception approach may utilize a single acquisition procedure or several sensors to constantly scan and monitor the surroundings, such as human-like vision, and other sensations. Collection, filtering, and dealing with raw data collected from a variety of sensors are all part of the process. In spite of extensive research into on-road driver assistance schemes and autonomous driving systems (including self-driving cars), methods established for organized traffic in a city environment may fail in an off-road setting due to the uncertainty and variety of unfamiliar conditions encountered^93^. The range, signal features, and detection conditions of a single sensor make it difficult to detect obstacles^94^. As a result, researchers and technologists are looking into multiple sensors and systems. The typical categories of sensors are Image-based sensors, Range-based sensors, and Hybrid sensors, while the most important methods of sensing are classification-based methods, probability-based methods, and inference-based methods^95^.

#### Localization and mapping (LM)

For almost 25 years, a continuous localization and mapping system has been a hot topic in the community of mobile robotics. The increasing focus on AVs has accelerated the research attempt with the assistance of automobile manufacturers^94^. The global navigation satellite system (GNSS) could be considered a solution to the problem of location; however, it was immediately demonstrated that this is not sufficient in and of itself^95^. Even though the accuracy constraints of any classical GNSS system are raised when ideally positioned base stations are employed with the kinematic GNSS, namely Real-Time Kinematic GNSS, availability continues to be a problem in this environment. The use of road infrastructures such as road markings or highway indications to guide a vehicle into a lane is another fundamental approach to localization and navigation^96^. These kinds of approaches, while limiting in their scope as the lateral positioning in the multi-agent traffic environment, are sufficient for contexts where the route can be clearly seen, such as highways. More complicated situations, particularly multiple-vehicle traffic environments, may not always give enough road data to locate a car accurately. Moreover, longitudinal position precision is more than crucial in straight, expressway-like situations^97^.

#### Object refinement (OR)

The quality of a self-driving system’s perception task significantly impacts its performance^98^. There has been a rise in the availability of scanners, like LiDAR, which allows for more precise depictions of the vehicle’s surroundings, resulting in safer systems. The results demonstrate that contemporary real-time object detection arrangements achieve high performance at the detection rate and the accuracy cost^99^. Hardware and software advancements are expected to lead to a better balance between run-time and detection rate object refinement (OR)^99^. However, current real-time OR networks are unsuitable for high-accuracy tasks like AVs visual perception^100^.

#### Maneuver illustration (MI)

The march toward more enhanced driver assistance systems and the advancement of AVs open up new opportunities for the safety system^95^. Improved MI methods may be developed due to increased information accessible in the vehicle regarding the surrounding traffic situations and the path ahead^97^. These systems will utilize this data for control stability during safety-critical maneuvers. In order to reduce the chance of a collision, such a method might adaptively trade-off between regulating the vehicle’s lateral, longitudinal, and rotational dynamics in order to achieve the best balance.

#### Vehicle health monitoring (VHM)

Many factors contribute to traffic fatalities and injuries, including poor vehicle maintenance, unfit drivers, careless driving, a lack of driving instruction, and poor decision-making when it comes to adhering to traffic regulations. Legislative bodies are also to blame for these accidents because they do not have the proper oversight in place. Developing a centralized intelligent VHM system appears to be an excellent answer to this situation^100^.

#### Cooperative perception (CP)

Precise localization is critical for navigation tasks in related fields such as AVs and intelligent transportation systems. The multi-vehicle perception process and control viewpoints are represented in^101^. Cooperative operations (CO) in multiple vehicle systems are intended to allow participants to trade sensed obstacles or perceived information with one another in order to broaden their sensory horizons, hence increasing their situational awareness and safety^102^. The concept of cooperative and non-cooperative accident-avoidance alert methods for overhauling or lane shifting assist and automatic lane shift is represented in another study^103^. Several research areas have looked into cooperative perceptions, incorporating sensor data handling, wireless network settings, and implementations of unified perceptions^104^. Certain researchers have used sensor fusion solutions to improve the reliability and precision of their data^105,106^. Authors in^67^ give a flowchart of the cooperative perception procedure in AVs.

**Table 4:** Perception aspects of MVCCA in AVs.

Refs.	Features Aspects									Pros	Cons
	EF	SF	LM	OR	MI	VHM	CP	SVCA	MVCA		
^91^	$\checkmark$	$\checkmark$	$\checkmark$	$\checkmark$		$\checkmark$		$\checkmark$		Presents the methods of sensor fusion.	Focused on a fully automated system and the mixed traffic was not in consideration.
^93^	$\checkmark$	$\checkmark$	$\checkmark$	$\checkmark$	$\checkmark$			$\checkmark$		Obstacle detection performance.	Multi-agent is not in consideration.
^107^			$\checkmark$		$\checkmark$	$\checkmark$	$\checkmark$	$\checkmark$	$\checkmark$	Discussed on algorithms for perception.	Empowered by DL algorithms only.
^94^		$\checkmark$	$\checkmark$	$\checkmark$		$\checkmark$	$\checkmark$		$\checkmark$	In off-road environments.	Obstacle avoidance methods.
^95^		$\checkmark$		$\checkmark$	$\checkmark$			$\checkmark$		Information-awareness by sensing.	Information used for the controller.
^96^		$\checkmark$	$\checkmark$	$\checkmark$		$\checkmark$	$\checkmark$	$\checkmark$	$\checkmark$	Discussed on environment perception.	Only simulation platforms.
^97^	$\checkmark$		$\checkmark$	$\checkmark$	$\checkmark$	$\checkmark$	$\checkmark$	$\checkmark$	$\checkmark$	Intention recognition utilizing mirror neuron.	Limited in-lane illustration process.
^98^	$\checkmark$	$\checkmark$	$\checkmark$	$\checkmark$	$\checkmark$		$\checkmark$	$\checkmark$	$\checkmark$	End-to-end approaches	Only software components.
^99^	$\checkmark$		$\checkmark$	$\checkmark$	$\checkmark$	$\checkmark$	$\checkmark$	$\checkmark$	$\checkmark$	Drawbacks of new automated systems.	Mixed traffic was not checked
^100^		$\checkmark$	$\checkmark$	$\checkmark$	$\checkmark$	$\checkmark$	$\checkmark$	$\checkmark$		AI-supported applications.	Discussed sensing systems.

*EF* Environment fusion, *SF* Sensor fusion, *LM* Localization and mapping, *OR* Object refinement, *MI* Maneuver illustration, *VHM* Vehicle health monitoring, *CP* Cooperative perception, *SVCA* Single vehicle collision avoidance, *MVCA* Multiple vehicle collision avoidance.

### Communication and cooperation

The second important fact of MVCCA in AVs mentioned in our taxonomy is communication & cooperation (CC). Over the past two decades, advances in robotics, navigation, sensing, computer vision, and high-performance computing have stimulated new automotive innovations, mainly through two streams. First, the automation of vehicles, where vehicle control functions autonomously without direct driver inputs (such as steering, throttle, and braking). Second, vehicle connectivity consists of different communication technologies^108^ for vehicles, such as V2V, V2I, and V2P^60^. Multiple vehicular communication research^109–113^ has been conducted to establish efficient and realistic cooperative communication systems. This work is dedicated to evaluating some existing reviews and journal papers in this phase as seen in Table 5. The possible impacts of vehicle communication and mutual awareness using the vehicular ad-hoc network (VANET) Veins simulator were explored by authors of^112^. The allocation of vehicle communications services through the use of value-anticipating networks was discussed in an article by^110^. In the survey, authors in^114^ reviewed communication security in a systematic literature review. The next subsections present some details of CC regarding AVs system.

#### Intra-vehicle networking (IVN)

In the AVs’ prospects, the IVN has some viable roles. Improved sensor technologies such as ranging and light detection, cameras, radar, and other sophisticated sensor technologies ushered in a new age in automated driving^115^. A consequence of the inherent constraints of these sensors is that AVs are more likely to make wrong decisions, which can result in fatal outcomes. At this stage, IVN technologies can compensate for sensor shortcomings and are more dependable, practicable, and efficient in boosting information interaction, resulting in improved AVs perception and planning skills and enhanced vehicle control^116^. Inter-vehicle communication is only possible if significant messages that increase safety can be exchanged quickly and efficiently. Many technical issues must be addressed to meet this requirement, involving low latency, high reliability, and guaranteed data rates^117^.

#### V2V communication

In MVCCA, cooperation and communication are essential. Recent developments in hardware, software, and communication techniques and the creation of diverse functions and standards have enabled the development of new technologies^115^. Vehicle-to-vehicle communication (V2V) technologies are now being integrated into automobiles, which can detect the driving behaviors of other participants. Sensors, communication technologies, and information systems are being unified into vehicles in order to create connected vehicle networks. In interconnected networks, vehicle-to-vehicle communication (V2V) is being applied to decrease traffic congestion, increase passenger safety, and effectively control vehicles on highways^95^. V2V communication generally delivers real-time traffic road state information (e.g., speed, acceleration, position) concerning the ahead vehicles. As part of an active traffic management method, I2V communication, on the other hand, primarily offers information on downstream traffic circumstances or local speed proposals^118^.

#### V2X communication

Like V2V communication, vehicle-to-everything (V2X) communications have potential in MVCCA. Also needed for new internet-of-things (IoT) applications, including intelligent transportation systems, self-driving cars, collision avoidance systems, and so on^119^. Vehicle IoT faces two major challenges. First, vehicle mobility causes network elements such as communication nodes, accessible wireless sources, and network intensity to shift spatially. Second, the problem is made even more complex because the communication network environment is changing over time. Vehicle IoT systems incorporate several network nodes and diverse wireless communication techniques, thus the network situation may change frequently. As a result, we must create a more intelligent communication system that can self-evolve^118^.

#### Driving behavior

Predicting and planning interactive behaviors in complex traffic situations presents a challenging task^116^. It is difficult to predict and arrange interactive behaviors in complex traffic scenarios. AVs struggle to assess conditions and eventually attain their own driving aims, particularly in situations involving multiple traffic participants who interact closely. It is complicated in a multi-participant setting, and typically, AVs suffer from potential driving policies to avoid single-vehicle incidents and collisions among multiple vehicles^119^.

#### Coordination and reasoning

The road environment, in general, contains a large number of participants. Cooperative multi-agent systems (MAS) are those in which multiple agents work together to complete tasks or optimize value through interaction^120^. The interactions between the agents, the complexity of a multi-agent problem, can rapidly increase with the number of agents or their behavioral sophistication^121^. Mapping, localization, and motion planning are three interconnected competencies that must be presented for a robot to operate well. A road or route between two entrenched configurations in a cost field must be calculated while considering mobility constraints, static obstructions, and dynamic obstacles^115^.

#### Cooperative perception sharing (CPS)

Recently, in cooperative autonomous driving, the CPS concept has garnered increasing attention as a plausible and feasible option to increase autonomous driving performance (safety, comfort, efficiency)^120^. There are two main types of technical approaches: centralized and distributed. Assuming the first scenario is, a single driver is a leader in keeping the other vehicles under control, including coordinating their driving. Each car intends to exchange local information with others, such as cooperative adaptive cruise control (CACC) and cooperative perception-based autonomous driving (CPAD)^122^.

#### Platooning

It is possible to use a vehicle platooning strategy in autonomous vehicles, which involves a lead vehicle and a group of vehicles following it^24^. Cooperative adaptive cruise control (CACC) governs the movement of the cars in platoon^115^. CACC is an upgrade to adaptive cruise control (ACC) that adds vehicle-to-vehicle (V2V) communications and consent to cars to travel in more compact and stable platoons than ACC permits. Most CACC systems necessitate communication between the next vehicle and the car in front of the platoon, depending on which is closer. This can be accomplished through the exchange of data on the vehicles’ longitudinal and lateral control systems (e.g., steering) along with management procedures that monitor platoon formation, driving maneuvers, and platoon disengagement^117^. Cooperative awareness messages (CAM) are used to exchange this data across connected vehicles.

**Table 5:** Communication and cooperation for MVCCA in AVs.

Refs.	Features Aspects									Pros	Cons
	IN	V2V	V2X	DB	CR	CPS	P	SVCA	MVCA		
^115^	$\checkmark$	$\checkmark$	$\checkmark$	$\checkmark$	$\checkmark$	$\checkmark$	$\checkmark$	$\checkmark$	$\checkmark$	Discussed network and communication.	Not consider the mixed traffic system.
^116^				$\checkmark$	$\checkmark$	$\checkmark$	$\checkmark$	$\checkmark$	$\checkmark$	Cooperative actions investigated.	Consider only lane change situations.
^117^			$\checkmark$	$\checkmark$	$\checkmark$			$\checkmark$		Technological problems illustration.	Not discussed cooperative aspects
^95^	$\checkmark$	$\checkmark$	$\checkmark$	$\checkmark$	$\checkmark$	$\checkmark$	$\checkmark$	$\checkmark$	$\checkmark$	Evaluate the crucial challenges.	Not consider mixed traffic.
^118^		$\checkmark$		$\checkmark$	$\checkmark$	$\checkmark$	$\checkmark$	$\checkmark$	$\checkmark$	Reviewed existing work.	Only as a form of social-AI capability.
^119^		$\checkmark$	$\checkmark$	$\checkmark$	$\checkmark$	$\checkmark$		$\checkmark$	$\checkmark$	Use cases of communication	In-feasibility of current technologies.
^120^		$\checkmark$		$\checkmark$	$\checkmark$	$\checkmark$	$\checkmark$		$\checkmark$	A novel vehicular communication based on coordinated driving protocol.	Limited to the driving conditions of lane-changing situations.
^121^			$\checkmark$	$\checkmark$	$\checkmark$	$\checkmark$	$\checkmark$		$\checkmark$	Evaluation and framing key facts.	Only some aspect of traffic flows.
^122^	$\checkmark$	$\checkmark$	$\checkmark$	$\checkmark$	$\checkmark$	$\checkmark$	$\checkmark$		$\checkmark$	Multi-vehicle systems.	Discussed a car-following model.
^24^		$\checkmark$	$\checkmark$	$\checkmark$	$\checkmark$	$\checkmark$	$\checkmark$		$\checkmark$	Summarizing coordination in AVs	Focused on particular conditions.

*IN* Intra-vehicle networking, *V2V* V2V communication, *V2X* V2X communication, *DB* Driving behavior, *CR* Coordination and reasoning, *CPS* Cooperative perception sharing, *P* Platooning, *SVCA* Single vehicle collision avoidance, *MVCA* Multiple vehicle collision avoidance.

The coordination of multiple autonomous agents raises several real-world issues. These studies use cooperative multi-agent systems models, whereby agents aim to achieve a common global goal^123^.

### Threat assessment (TA)

Threat assessment determines the nature of a situation and assists in the secure operation of intelligent vehicles. As MVCCA is intended for threat assessment, several critical metrics could be established. It is essential to decide on an appropriate critical metric for resolving certain driving and navigation issues in various driving situations. In their article^124^ authors attempted to use an integrated algorithm for predicting obstacles and estimating the state of a self-driving vehicle. The authors claim in their article^125^ that the TA system performance will be stimulated by a decision-making scheme that will define the vehicle’s next plan of action.

Essential metric^126^ classified them into five groups: kinematics-based metrics, potential field-based metrics, time-based metrics, unexpected driving measures-based metrics, and statistics-based metrics. In the paper^127^ authors listed a large set of data with more than 250,000 kilometers of driving data for estimating the frequency of collisions with EVT (extreme value theory). Vision-based road safety identification techniques were reviewed by^32^. In their article^128^ they pointed out that automated car systems were first disassembled into vehicle components and transport infrastructure components to identify the risks. Many reviewers reviewed many pieces of literature on the tremendous potential of evolving automotive technology for safety and the environment. Table 6 refers to threat and presents the evaluations of some papers focusing on threat assessment as well as potential features, and the upcoming sections are the in-depth discussion of some facts mentioned in the proposed taxonomy.

#### Threats of in-vehicle protocol

Due to the lack of human control, it is critical for AVs to perceive the ambient situations precisely when cruising on the road^125^. AVs require a variety of sensors, including GPS, ultrasonic sensors, light detection and ranging (LiDAR), and millimeter-wave (MMW) radar. Sensors enable AVs to perform tasks such as sensing, obstacle/pedestrian recognition, collision avoidance, navigation, and more. Given the great reliance on sensors, it is possible that if they are blinded, or even intentionally managed, lethal disasters may result in^126^. The privacy of in-vehicle network connections, such as LIN, CAN, or FlexRay, must be taken into consideration^129^.

#### Driving comfort (DC)

The smoothness and consistency of a path are the two key parameters impacting DC in a multi-agent autonomous driving technique^130^. An uneven road may cause occupant discomfort or even wheel slippage, reducing the vehicle’s stability. The smoothness factor is gathered at the present planning phase to minimize chain collisions, but it cannot prevent the construction of a path that is substantially different from a path generated in a prior step^131^. If the difference between the current step’s path and the prior step’s path is too great, an abrupt transition will occur. Path consistency must be examined to avoid this situation^127^.

#### Collision risk prediction (CRP)

Early detection of dangerous conditions and proactive responses aid in maintaining appropriate safety distances. However, unexpected, unpredictable situational changes, dangerous maneuvers, and crashworthiness persist as an important aspect of vehicle protection, helping to reduce the severity of crashes^125^. The following are criticality measurements for regular automated driving. The Time-to-X-Metrics^126^, such as Time-to-Brake^132^, Time-to-Collision^127^, and Time-to-Steer, are probably the most well-known criticality metrics. In their direct relationship to human reaction time, these measurements are frequently utilized in assisted driving. However, they mainly concentrate on collision avoidance using imprecise motion forecasts based on constant velocities and do not take into account unpredictable environmental data. Work has been conducted on a vehicle cooperative collision avoidance (CCA) approach using the dedicated short-range communication (DSRC) for the V2V^133^. A unique decentralized and cooperative policy for collision-free motion coordinating of non-holonomic AVs was developed for the study.

#### Collision mitigation (CM)

The research direction presented three techniques for single and MVCA, as well as CM: (a) front collision indication; (b) front collision avoidance by decelerating and navigation; and (c) a combination of (a) and (b)^129^. The majority of earlier collision avoidance research did not see an improvement in V2V communication for MVs coordination. The time delay between sensor recognition and driver/agent reaction will accrue and spread upstream in MVs. If they follow each other closely, which is common on freeways, this is likely to result in numerous car collisions, especially if the first vehicle does emergency braking^134^. If the ego agent/vehicle is too close to the front agent/vehicle, steering may not be effective. Furthermore, if participant vehicles are on both the lanes left and right, steering could result in more serious collisions. A scheme is described as a group of agent vehicles that are longitudinally connected. If the velocity and distance of two neighboring vehicles in the same lane satisfy certain parameters, they are considered linked. Intuitively, if the leading car brakes, the following vehicle must take prompt action for safety. The time gaps used for realistic road driving are typically $1.4\ \sim \ 2.1s$, although some are as low as 0.4*s*. As a result, most vehicles in the same lane are grouped together in some way^132^.

#### Dynamic and static threat assessment (DSTA)

Though vehicular localization is required for multi-vehicle collision avoidance, several methods presume flawless sensing and positioning and instead use global positioning via an overhead tracking camera to avoid local procedures^134^. However, in order to conduct local collision avoidance accurately in a realistic environment, a vehicle must be able to estimate its own and other agents’ and humans’ positions without the use of external tools^135^. Furthermore, in a real-world setting, MAS requires strategies to deal with uncertainty in their own positions as well as the positions and potential actions of other agents^125^.

#### Uncertainty assessment (UA)

In a complex traffic environment, situation assessment is essential for a good vehicle safety method^132^. An illustration of the contemporary methods of ADAS shows that: i) the human-driving procedure involves observation, driver intention, and driving action submodules; and ii) the ADAS procedure contains detection and estimating, threat-assessment, decision-making, and instinct functions^132^. Indeed, ADAS operations are intended to be an idea just like the human-driving manner, and significant progress has been achieved in broadening the variety and difficulty of situations handled today. In the presence of several vehicles, a key theoretical difficulty remains how to correctly discern a safe driving behavior from a hazardous one, highlighting the importance of UA in AVs systems^133^.

#### Threat assessment strategies (TAS)

As vehicles become more automated, they must be able to analyze risks and evaluate situations in real time. Driver-less vehicles in this scenario should be able to assess risks in a dynamic environment in order to make informed decisions and adjust their driving behavior accordingly^126^. To avoid crashes, we must use a risk estimator that takes into account risk indicators such as (1) the driver’s state, (2) the conduct of other vehicles, and (3) the weather circumstances^131^. The collision avoidance (CA) system is one of the most important components of ADAS. Threat assessment, path planning, and TAS are commonly included in a suitable CA architecture. Using a combination of these methodologies, there are numerous approaches to construct exact CA architecture^127^.

**Table 6:** Reviewed up-to-date papers according to SVC and MVCs threat assessment in AVs.

Refs.	Features aspects									Pros	Cons
	TVP	DC	CRP	CM	DSTA	UA	TAS	SVCA	MVCA		
^125^	$\checkmark$	$\checkmark$		$\checkmark$		$\checkmark$	$\checkmark$	$\checkmark$	$\checkmark$	Comprehensive CA system highlighted.	Reviewed an introductory idea.
^126^	$\checkmark$	$\checkmark$	$\checkmark$	$\checkmark$		$\checkmark$	$\checkmark$	$\checkmark$		Comparative review of critical metrics.	Considering only three typical scenarios
^129^		$\checkmark$	$\checkmark$	$\checkmark$	$\checkmark$		$\checkmark$	$\checkmark$		Continuous real-time risk assessment.	Decision made on incomplete data.
^134^	$\checkmark$		$\checkmark$	$\checkmark$	$\checkmark$	$\checkmark$	$\checkmark$	$\checkmark$		Human-centered risk assessment.	Not applicable for motion control.
^133^	$\checkmark$	$\checkmark$			$\checkmark$	$\checkmark$		$\checkmark$	$\checkmark$	Analysis the effect of warning system.	Only in simulation environments.
^136^	$\checkmark$	$\checkmark$	$\checkmark$		$\checkmark$		$\checkmark$	$\checkmark$		Real-time NL collision prediction.	Interaction-aware model.
^132^	$\checkmark$	$\checkmark$	$\checkmark$	$\checkmark$		$\checkmark$		$\checkmark$	$\checkmark$	Identify the harmful situation.	Did not solve the problem.
^135^		$\checkmark$		$\checkmark$	$\checkmark$	$\checkmark$	$\checkmark$	$\checkmark$	$\checkmark$	Proposes a proactive cyber-risk model.	Only concerning the cyber-risk assessment.
^137^	$\checkmark$		$\checkmark$	$\checkmark$		$\checkmark$	$\checkmark$	$\checkmark$		Analysis of threat-assessment methods.	Cover only single-behavior threats.
^138^	$\checkmark$	$\checkmark$		$\checkmark$	$\checkmark$	$\checkmark$		$\checkmark$	$\checkmark$	Survey of existing methods.	Study marginally.

*TVP* Threats of in-vehicle protocol, *DC* Driving comfort, *CRP* Collision risk prediction, *CM* Collision mitigation, *DSTA* Dynamic and static threat assessment, *UA* Uncertainty assessment, *TAS* Threat assessment strategies, *SVCA* Single vehicle collision avoidance, *MVCA* Multiple vehicle collision avoidance.

### Decision making

The current autonomous driving system is prone to rear-end collisions, and it is a typical cause of MVCs. An optimum decision-making strategy is needed to prevent this type of collision. Authors of^139^, examine fleet management issues in single and multiplayer transportation networks. In the article^140^, authors focused on their annual study of recent trends in AVs driving decision-making planning. This review discussed some of the latest findings related to various areas of AVs decision-making and planning in Table 7. A valuable review of the decision-making and control systems of AV is available^141^.

Using a rigorous mathematical framework, authors^142^ formulate and discuss the optimization algorithm for the solution and examine the main details of the implementation of the multi-vehicle motion planning problem. In the article^124^, the authors propose a new way of thinking in which agents learn collision as a single agent and then avoid multiple collisions by reversing the trained policy. Major research using quadratic mixed-integer programming (MIQP) has been conducted^143^, with others implementing B-splines^144^, polynomials^145^, elastic bands^146^, and potential fields^147^, in route planning strategies^148^. Contemporary research takes into account the problem of route planning for a single vehicle when multiple vehicles are present in a traffic environment. The following subsections are the discussion about the decision-making aspects of multiple participants’ environments in AVs.

#### Cooperative motion planning (CMP)

CMP for automated cooperative collision avoidance in a multiple-vehicle setting is a possible future solution to improve traffic safety. This method necessitates a real-time motion analyzer that calculates several cognitive vehicles’ cooperative moves. Path planning is a computationally demanding operation, the planner’s computing time must be balanced against the solution’s efficiency^139^. Automatic involvement of this support system in dangerous scenarios involving many vehicle accidents. Human drivers have a long response time and few opportunities to organize their actions with many other drivers, they are frequently unable to initiate the right actions^140^. A fundamental requirement for the designed method is planning cooperative moves that avoid or lessen accidents.

#### Cooperation and interaction (CI)

Cooperative MAS are processes where several agents work together to solve problems or maximize utility through the interactions between the agents. The complexity of a multi-agent issue can rapidly increase as the number of agents or their behavioral sophistication increases. Due to the difficulty in programming solutions to MAS problems, machine learning approaches to facilitate the search and optimization process are gaining popularity. Typical solutions^149^ for dealing with those specific maneuvers are rule-based methods that use some notion of time-to-collision^141^ to ensure that they are only executed if there is more time in the worst-case scenario. Due to the lack of explanation of the situation, these options led to overly cautious behavior. It was suggested that machine learning methods, such as partially observable Markov decision processes or deep learning techniques, be used to infer the intentions of other drivers^150^. However, training machine learning algorithms of this type usually necessitates simulated environments, behavioral simulation of other drivers is crucial^139^.

#### Collective decision-making (CDM)

Various CDM procedures have been created in MAS research to obtain consensus over the agents’ collected preferences. In automotive applications, voting processes have been used to establish agreements in car-sharing^149^, platooning, and leader election in decentralized intersection control^151^. It is able to brake properly, not like the driver’s late or poor reply to risk conditions, reducing the vehicle’s speed and the severity of the crash. As a result, designing accurate and efficient low-level automated braking control methods or high-level control depending on coordinated techniques is a huge technical issue. Conventional control techniques, like constant time headway (CTH), constant spacing (CS) policy, and sliding mode control (SMC)^139^, have a limited ability to adapt to changing driving environments in reliable and realistic decisions when CAVs coexist with traditional driver-controlled vehicles^140^.

#### Autonomous braking decision (ABD)

In accidents, autonomous braking via accurate vehicle decision-making is crucial, especially in the initial phases of AVs technology^141^. ABD is completely dependent on the automated braking function (ABF), which is one of the AVs safety cores technologies^151^. It can successfully brake, as opposed to the driver’s reaction to dangerous situations, which is either too late or inadequate, reducing the vehicle’s speed and the accident’s repercussions. The intelligent control system, assisted by the present advancement of AI, makes decisions based on the present environment and continuously learns and adapts to it^152^.

#### Trajectory coordination (TC)

One of the concerns in autonomous multi-robot systems is how to avoid crashes between separate robots. Finding a coordinated trajectory from beginning to goal for all robots and then allowing the robots to follow, which was TC, is one method to solve this challenge (TC)^153^. *Classical prioritized planning*, in which robots plan sequentially one after the other, is a frequently used practical method for discovering such coordinated trajectories^152^. This method has been demonstrated to be effective in practice, but it is unfinished, and it has not yet been properly assessed under what conditions the method is certain to succeed. Furthermore, prioritized planning is a centralized algorithm, it is unsuited for decentralized multi-robot systems and the avoidance of chain collisions^154^.

#### Longitudinal and lateral constrains (LLC)

In collision avoidance decision-making, optimization methods simultaneously defeat decentralization effects^142^. They use longitudinal and lateral constraints (LLC) to optimize a cost function concerning a collection of states and the input^155^. Several real-time motion planning issues are non-convex, optimization problems may become stuck in local minima and become computationally inefficient. Optimization issues can become stuck in the local bare minimum and inefficient to solve many motion planning problems that are non-convex. Deploying the optimal collision avoidance decision-making approach in both single and multiple vehicle collisions is a system need^153^.

**Table 7:** Various areas of autonomous vehicle decision-making and planning focused on MVCCA in AVs.

Refs.	Features aspects									Pros	Cons
	CMP	CI	CDM	FM	ABD	TC	LLC	SVCA	MVCA		
^139^	$\checkmark$	$\checkmark$	$\checkmark$	$\checkmark$		$\checkmark$	$\checkmark$	$\checkmark$	$\checkmark$	Framework for multiple players.	A computational technique.
^140^	$\checkmark$	$\checkmark$	$\checkmark$	$\checkmark$		$\checkmark$	$\checkmark$		$\checkmark$	Behavior-aware planning.	Considered particular cases and did not attempt to the universe the system.
^149^		$\checkmark$	$\checkmark$		$\checkmark$	$\checkmark$	$\checkmark$		$\checkmark$	A Generic Mixed-Integer Formulation.	Not considering the multi-agent cases.
^150^	$\checkmark$		$\checkmark$		$\checkmark$	$\checkmark$	$\checkmark$		$\checkmark$	Cooperative conflict resolution.	Computational complexity is high.
^151^		$\checkmark$			$\checkmark$	$\checkmark$	$\checkmark$	$\checkmark$	$\checkmark$	Cooperative trajectory planning for MV.	Arbitrary road geometry.
^142^		$\checkmark$	$\checkmark$	$\checkmark$		$\checkmark$	$\checkmark$		$\checkmark$	Motion planning for multiple vehicles.	Exclusively by preplanning step.
^154^	$\checkmark$		$\checkmark$		$\checkmark$	$\checkmark$	$\checkmark$		$\checkmark$	Tracking and decision-making.	Particularly the implementation of stochastic policy.
^152^	$\checkmark$	$\checkmark$		$\checkmark$	$\checkmark$	$\checkmark$	$\checkmark$	$\checkmark$		Review on motion planning.	Highway geometric planning.
^155^	$\checkmark$	$\checkmark$		$\checkmark$	$\checkmark$	$\checkmark$		$\checkmark$	$\checkmark$	Decision-making in multi-agent.	Lead extreme time consumption.
^153^	$\checkmark$	$\checkmark$		$\checkmark$	$\checkmark$	$\checkmark$		$\checkmark$	$\checkmark$	Decision-making highly AVs.	May need more research.

*CMP* Cooperative motion planning, *CI* Cooperation and interaction, *CDM* Collective decision making, *FM* Fleet management, *ABD* Autonomous braking decision, *TC* Trajectory coordination, *LLC* Longitudinal and lateral constraints, *SVCA* Single vehicle collision avoidance, *MVCCA* Multiple vehicle cooperation and collision avoidance.

### Vehicle control for MVCCA in AVs

According to the proposed taxonomy, motion planning, decision-making, and vehicle control are critical for multi-agents to navigate in their environment. In this section, we review a set of the most relevant review articles and journals from the perspective of both single and multiple automated vehicle traffic environments. We evaluate the main features as well as their decision-making limitations in additional review papers in Table 8. In the coming subsections, we discussed some details of every aspect of our taxonomy.

In order to take into account the prevention of collisions and the mitigation of their impacts in a multi-vehicle collision situation, it is only appropriate to take into account a longitudinally coupled structure evolving of nearly followed vehicles. Coupled refers to two adjacent agents in the same lane if such criteria are met jointly by their speed and distance^156^. Authors of the article^153^, examined the existing controller system in a mixed traffic system and concluded that the human driver car should be accurately modeled as an essential agent in shared drivers’ vehicle control systems in terms of cognitive processes, control mechanisms, and decision-making processes. In this context, the swarm intelligence algorithms are recently getting popularity to solve this complex problem and by this method, numerous types of research have been conducted to optimize the driving decision policies to get the optimum outcomes^157^. Considering multiple agent traffic patterns, in paper^158^, authors reviewed and demonstrated an architecture for IVDC (integrated vehicle dynamics control) for a quicker and more versatile design to help car manufacturers and suppliers.

#### Obstacle avoidance (OA)

The most difficult task in autonomous driving systems is avoiding both static and moving obstacles, which is still hampered by optimal policy procedures^159^. The problems arise from an integrated process of detecting and interpreting the surroundings and impediments, as well as the production of appropriate behaviors^160^. As a result, having a superior control strategy that can drive in an urban setting without colliding with other vehicles and objects is desirable^161^. The majority of current research does not concentrate on the sub-task of obstacle avoidance (OA) in specific driving scenarios. However, on a normal road, other vehicles or obstructions can have a significant impact on the car, therefore OA is a problem that AVs must overcome. Cars can collect data and route information, such as road conditions and location estimations of static and dynamic objects, and use it to forecast actions taken by other vehicles and infrastructure in real-time^162^.

#### Geometric model (GM)

The recognition of moving objects is frequently required in the first step of computer vision applications^159^. Background subtraction is used to segregate the foreground from the background. However, the main objective is to use background removal techniques in research in real-world applications such as traffic surveillance^163^. However, a review of the literature reveals that there is frequently a detachment between current approaches utilized in real-world applications and current techniques in basic research. Furthermore, the videos assessed in substantial-level datasets are not comprehensive, as they only reflect a portion of the full range of issues encountered in real-world applications^164^. For example, for image data synthesis, a visual structure is applied to produce an estimated geometric representation of an object, whether the image input is static. The second example enables the creation of an image-based human model that may be utilized for optical motion capture^165^.

#### Kinematic model (KM)

To detect unanticipated variations in participant and ego behavior, a kinematic framework based on the physical phenomena of kinematics is used^158^. The KM is also used to detect unexpected deviations by leveraging information from the leader vehicle, which is directly conveyed and monitored by the leader’s nearby cars and supporting infrastructure. The KM is reliable, but not optimal, in particular in the MVCCA aspect^160^.

#### Dynamic model (DM)

The majority of technical obstacles arise from the unpredictable environment in which AVs operate, such as road and weather conditions, perceptual and sensory input data mistakes, and ambiguity in pedestrian and agent vehicle behavior^27^. A robust AV control algorithm should account for many sources of uncertainty and generate measurable safe control rules. Furthermore, algorithms that follow precise security measures can aid legislators in handling AV-related legislation difficulties, such as insurance policies, and ultimately persuade the public to accept AVs on a large scale^162^.

#### RL based control

The reinforcement learning (RL)-based automated decision-making strategies function relatively well enough in the autonomous driving system’s ongoing learning and feedback feature. Researchers have fantastic solutions for enhanced autonomous decision-making and control for AVs. In the article^165^, authors propose a particular precise deep Q-network-based automatic braking system to avoid vehicle-pedestrian collisions (DQN). Subsequently, in the article^163^ authors created a cooperative adaptive cruise control (CACC) automobile controller based on RL. Recently, the authors, in^165^, proposed a framework for CACC systems based on supervised reinforcement learning (SRL). However, authors in^166^ proposed a considerable method to overcome the coordination problem in autonomous driving using multi-agent reinforcement learning (MARL).

#### Cooperative control (CC)

The majority of studies on multi-AV control fall under cooperative coordination^158^. In other words, AVs are expected to connect for global traffic information and optimize a common goal of improving traffic flow. In multi-robotic applications, cooperative control has received a lot of attention^167^. For a group of robots with a centralized aim to achieve a task collectively, swarm intelligence, formation control, and consensus control have all been widely employed, as has multi-AV control^162^. A centralized controller or planner coordinates the movement of vehicles in cooperative multiple-vehicle systems to achieve a shared goal, like collectively stabilizing traffic flow and smoothing traffic jams, optimizing driving comfort, or improving fuel efficiency^165^.

#### Non-cooperative control (Non-CC)

A MAS is a collection of vehicles that interact in a shared environment that they detect with sensors and act on with actuators^162^. Distributed control, robotic teams, resource management, data mining, collaborative decision support systems, and other disciplines use multi-agent systems^166^. They may emerge as the most natural way of looking at systems or provide an alternative viewpoint on systems previously thought to be centralized. Robotics, telecommunications, distributed control, and economics are just a few fields where multi-agent networks are finding use. Due to their complexity, many tasks that arise in these fields are challenging to solve using pre-programmed agent actions. However, the agents must find a solution independently. A substantial portion of multi-agent learning research focuses on reinforcement learning techniques^165^.

**Table 8:** According to latest works the major features of vehicle control for MVCCA in AVs.

Refs.	Features Aspects									Pros	Cons
	OA	GM	KM	MD	RLBC	CC	NC	SVCA	MVCA		
^159^	$\checkmark$	$\checkmark$	$\checkmark$	$\checkmark$		$\checkmark$	$\checkmark$	$\checkmark$		A nonlinear vehicle models.	Focused only on modeling.
^167^	$\checkmark$	$\checkmark$			$\checkmark$	$\checkmark$		$\checkmark$		Vehicle control DL methods.	Deep learning methods only.
^158^		$\checkmark$	$\checkmark$	$\checkmark$			$\checkmark$	$\checkmark$		Reviewed control techniques.	Path tracking concepts.
^27^	$\checkmark$	$\checkmark$	$\checkmark$	$\checkmark$		$\checkmark$		$\checkmark$		Trajectory motion controller.	Verified only in simulation (Carsim).
^163^	$\checkmark$					$\checkmark$			$\checkmark$	Cooperative navigation algorithm.	Not guaranteed to deadlock avoidance.
^160^	$\checkmark$	$\checkmark$		$\checkmark$			$\checkmark$	$\checkmark$	$\checkmark$	Cooperative approach for multi-agent.	Strategies verified only in simulation.
^161^	$\checkmark$	$\checkmark$		$\checkmark$			$\checkmark$	$\checkmark$		Investigate the trajectory modeling.	Multiple vehicles are not considered.
^165^	$\checkmark$	$\checkmark$		$\checkmark$		$\checkmark$		$\checkmark$	$\checkmark$	Computational techniques.	Intermodal fleet planning.
^162^	$\checkmark$	$\checkmark$		$\checkmark$	$\checkmark$	$\checkmark$		$\checkmark$		Test and compare decision and control.	Simulate interactive driver behavior.
^166^	$\checkmark$				$\checkmark$	$\checkmark$			$\checkmark$	Vision-based DL and RL methods.	The perception input was static.

*OA* Obstacle avoidance, *GM* Geometric model, *KM* Kinematic model, *DM* Dynamic model, *RLBC* RL based control, *CC* Cooperative control, *NC* Non-cooperative control, *SVCA* Single vehicle collision avoidance, *MVCA* Multiple vehicle collision avoidance.

## Conceptual framework of MVCCA

### Proposed framework

**Figure 7:**
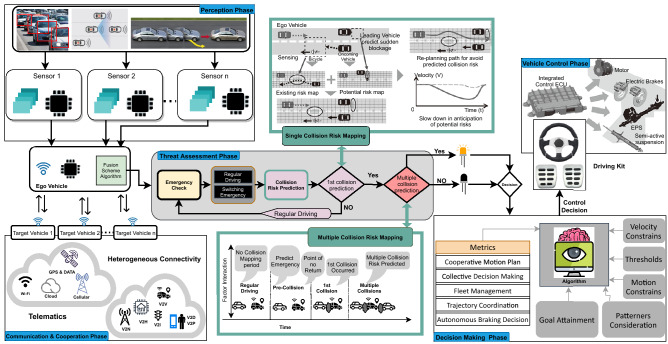
Proposed AI-enabled conceptual framework for MVCCA in AVs.

According to the existing research works, we have developed our taxonomy to solve the MVCC problem. Moreover, illustration of current automated vehicles (AVs) research work has shown that multiple factors and indicators causing vehicle crashes are not thoroughly defined, categorized, or modeled in an embracing context that can be incorporated into applications. Research on multiple agents in AVs is more complex and undiscovered until now. We reviewed contemporary research in detail and created a novel approach to collision avoidance strategies in AVs. We proposed an AI-enabled conceptual framework that has five phases. Due to focusing on the decision-making phase, we also proposed a reinforcement learning-based model to make a perfect driving decision for avoiding chain collisions or mitigating the chain collision severity. We define a specific threat assessment phase containing three distinct levels of risk. The first level is the regular driving situation, the second level is a bit careful driving condition where the ego vehicle will predict only one participant vehicle near its minimum distance. Finally, when the ego vehicle detects more than one participant in the range of the standard distance, it will make an extra careful driving situation. We need to train our model using a trial and error process to adopt our kinematic constraints^168^. Figure 7 shows the proposed conceptual framework for MVCCA in AVs, and in the following sections, we will discuss briefly all five phases.

#### Perception phase

From the review works in *section* 4, due to the mitigation of multiple vehicle collisions, the perception of a multiple-agent environment can obviously be more sophisticated than regular driving. Utilizing the segmentation and detection algorithms, we divided the risk prediction phase into two distinct stages, where our risk index for multiple vehicle collisions is normally zero when only one road user is detected. It will become high when it detects two or n number of partners surrounding itself. The risk index will reach a high level when the first crash occurs for any unpredictable reason; in this critical situation, we suggest the vision-based supervised learning perception methods that are now very popular in the AI community. In the proposed framework, a local processing scheme could be suggested to achieve highly accurate localization. Map-supported localization algorithms are used to conduct the local features. In particular, we defined the simulations by considering the prominent method as simultaneous localization and mapping (SLAM). The aim of framing SLAMi as a Bayesian filtering problem is to estimate the joint posterior probability,

1 $$\begin{aligned} P(x_1:k, b|s_1:k, q_1:k-1) \end{aligned}$$

where *b* is the map and $x_1:k= x_1,\ldots x_k$ the robot trajectory given its sensor measurement $s_1:t= s_1,\ldots s_t$ and the device inputs $q_1:k-1= q_1,\ldots q_(k-1)$. In this group, Kalman filter is a common method. The RBPF shows the trajectory of the vehicle and the corresponding map and factories, the probabilities are as follows:

2 $$\begin{aligned} P(x_1:k, b\,|s_1:k,q_1:k-1)= P(b\,| x_1:k,s_1:k) .P(x_1:k|s_1:k,q_1:k-1) \end{aligned}$$

Here, the posterior probability is calculated by a particle filter. $P(x_1:k,\,b|s_1:k,q_1:k-1)$ in which the previous distribution is derived from the odometry of participant vehicles and refined with sensor interpretations in a multi-agent dynamic environment.

#### Communication and coordination phase

This is the second phase of our conceptual framework for MVCCA in AVs. In the multiple-agent traffic environment, platooning, lane merging, and truck overtaking are extreme cooperative coordination situations with patterns of road users. In some critical conditions such as after the occurrence of the 1st crash, despite the high risk of 2nd, 3rd, or multiple crashes, there are situations that are not clearly or unproductively controlled and where cooperation is required to avoid chain collisions. Therefore, as far as the communication medium is concerned, two high standards would be set with 5G, a promising choice for the system. If a unified level of preparation is preferred, C-V2X has to be chosen for backend communication. IEEE802.11p would also be sufficient for decentralized planning with coordination. A combination of both methods is, of course, possible again, whereby routing information is obtained from a central planning level via c-V2X, and maneuver planning could be organized via V2X locally.

#### Threat assessment phase

Our proposed conceptual threat assessment phase will estimate the situation’s criticality and aid in ensuring safety in the automated traffic system. Two critical metrics have been suggested (see Fig. 7) for threat assessment, namely single collision, and multiple collision, and the selection process of the critical metric must be good for specific driving actions in diverse driving environments. The previous *section* "Challenges and issues of MVCCA" provides a comparison of vital indicators, with an emphasis on real-time automated driving strategies. According to that comparison, we would like to suggest the RL-based techniques that are required by automated systems operating in complex, dynamic, and interactive environments that generalize the interactions with multiple traffic participants to unforeseen circumstances and timely rationales. We presented an in-depth framework in our previous work in^169^, where we utilized the critical condition prediction technique depending on a recurrent neural network-based technique.

#### Decision-making and vehicle control phase

In our proposed conceptual framework, the last two phases namely decision-making and vehicle control are the most concentrated phases. From the previous sections, we can say that the AVs’ decision-making process must deal with a diverse set of situations, communicate with other traffic participants, and should be able to take into account a set of sensor information from the environment as well as the uncertainty. It is impossible to manually predict all circumstances that may arise and code a suitable behavior. Therefore, considering methods focused on machine learning to train a decision-making agent is convincing. A desired feature of such an agent is that it does not only deliver a recommended decision but also measures the uncertainty of the decision in question. Deep neural networks (DNNs) are a common artificial intelligence technique for learning after large quantities of data with little human input or without any human interactions (i.e., RL methods). The developed agents are learned and can operate in unpredictable, broad, and stochastic contexts, as revised. The agent has been particularly trained by the effective way of a combination of Reinforcement Learning (RL) and Deep Reinforcement Learning (DRL)^170^. We proposed a multi-agent DRL based on an ideal driving strategy for avoidance or mitigating multiple collisions. The following are the details of the proposed MVCCA strategies decision-making and vehicle control mechanism.

*RL Method:* An RL method may learn how an agent should behave in order to maximize the predicted cumulative rewards by interacting with the environment for a specific activation in a specific state. Existing RL algorithms are categorized into two key types: value-based and policy-based methods. Value-based RL methods, use neural networks to solve value functions. The main advantage of policy-based RL methods is in the phase of optimization, which can directly improve policy optimization while remaining stable over time during approximation. Regarding our objectives which we defined in previous sections, here, we proposed a policy-based RL approach to address multiple collision avoidance issues. The general form of loss function for RL policy updating, in a stochastic RL where ${\hat{{\mathbb {E}}}}_t$ is the expectation policy ${\pi }_\theta$, and at time step *t*, ${{\hat{B}}}_t$ is an estimator of the function and the mathematical expression is,

3 $$\begin{aligned} {\mathscr {L}}(\theta )=\ {\hat{{\mathbb {E}}}}_t\left[ \log {\pi _\theta (\alpha _t|s_t){{\hat{B}}}_t}\right] \end{aligned}$$

Although performing several optimization steps on this, ${\mathscr {L}}(\theta$) (loss function) can seem appealing and straightforward, all the factors may pose problems, such as the prevalence of sample inefficiency, the exploration and exploitation trade-off, and the learned policy carriages unwanted high variance. In practice, this frequently leads to major policy updates, and it will be harmful at a future time step of a training episode because it can change the distribution of observation and reward. In contrast, it is important to use an actor-critical mechanism for modifying a policy that can combine the advantages of conventional value-based and policy-based approaches in the loss function $${\mathscr {L}}(\theta )\ $$ Proximal Policy Optimization (PPO) and Trust Region Policy Optimization (TRPO) are examples of policy based algorithms. For simpler implementation, PPO is more convenient than others because of its less computational cost. PPO offers paired substitute loss function, a feature that can be combined as a policy substitute and an error term of value-function, and can be expressed as follows:

4 $$\begin{aligned} {\mathscr {L}}_t^{goal+UF+P}(\theta )=\ {\hat{{\mathbb {E}}}}_t[{\mathscr {L}}_t^{goal}(\theta )\ -\ K_1{\mathscr {L}}_t^{UF}(\theta ) +K_2P\left( \pi _\theta |(p_t))\right] \end{aligned}$$

where, the paired substitute goal is ${\mathscr {L}}_t^{goal}(\theta )$, $K_1$, $K_2$ are coefficients, ${\mathscr {L}}_t^{UF}$ is the value function’s squared error loss ${(U}_\theta (p_t) -U_t^{targ})2$, and the loss of entropy denoted by *P*. Specifically, the paired substitute goal is $${\mathscr {L}}_t^{goal}(\theta )\ $$ takes the form as,

5 $$\begin{aligned} {\mathscr {L}}_t^{goal}(\theta )=\ {\hat{{\mathbb {E}}}}_t\left[ min(r_t(\theta ){{\hat{B}}}_t,goal(r_t(\theta )),1-\varepsilon ,1+\varepsilon ){{\hat{B}}}_t\right] \end{aligned}$$

where, $\varepsilon$ is hyperparameter,${\ r}_t(\theta )$ is probability ratio of ${\ r}_t(\theta )=\ \pi _\theta (\alpha _t|s_t)/\pi _{\theta _{old}}(\alpha _t|s_t)$. The probability ratio *r* is the goal objective whose paring is at $1- \epsilon$ or $1+ \epsilon$, and it depends on whether it is a positive advantage or a negative advantage, forming the paired goal target as well as the ultimate goal after multiplying ${{\hat{B}}}_t$, is the approximate advantage. In contrast to the unpaired version, also known as the conservative policy iteration algorithm’s loss function, the ultimate value of ${\mathscr {L}}_t^{goal}(\theta )$ takes the minimized value of this paired goal objective and unpaired goal objective $r_t(\theta )$, essentially avoiding a broad policy update.

The PPO algorithm typically utilizes a stable length-*N* trajectory segment that runs the *N*-time steps of policy in each iteration, and each *M* parallel actor collects data at each time step. It uses a simplified version of the generalized gain estimate, which looks like this:

6 $$\begin{aligned} {{\hat{B}}}_t=\delta _t+(\gamma \lambda )\delta _{t+1}+.......+{(\gamma \lambda )}^{N-t+1}(\delta _{N-1}) \end{aligned}$$

where the discount factor is $$\gamma \ $$ and $\gamma \ and\ \delta _t\ =\ r_t+\ \gamma \ V(s_{t+1}-V(s_t)$. Then the loss $${\mathscr {L}}(\theta )\ $$) is created by *PPO* and *SGD* is the optimizer with mini-batch , for epochs *K* on these time steps *MN* of data.

### Proposed control learning model

**Figure 8:**
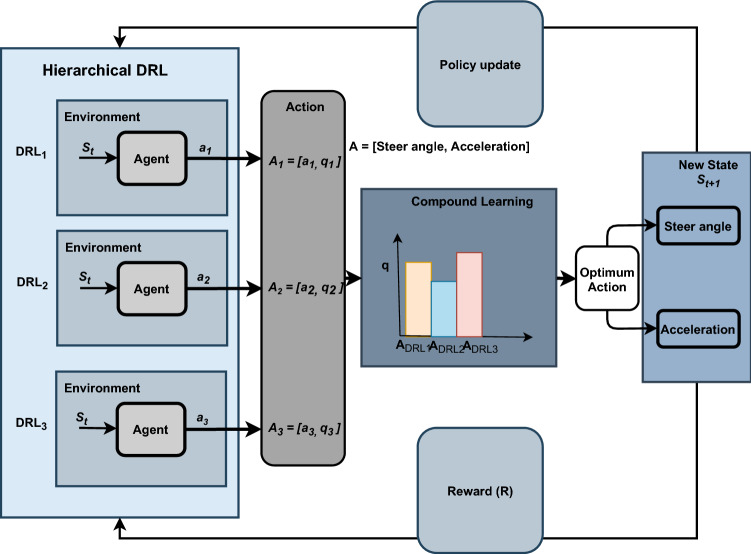
Proposed decision-making model for avoid MVCs.

The decision-making model for vehicle control in the multiple agent traffic environment is defined as a hierarchical DRL method that relies on three DRL techniques. The best driving policy or set of actions will be determined based on the DRL technique with the highest Q value. Three DRL techniques will be used to figure out which one has the highest Q value. The results of the DRL control actions can be optimized using compound functions by comparing the sets of actions and value functions based on the most recent states of their respective objective functions. When all the Q values from the three different DRL schemes are compared to one another, high-performing value function targets are identified, and a new set of value functions that are based on all the learning control functions is generated. If a high value was chosen, this would lead to an increase in the value of objective functions that involve preference comparisons, which in turn would lead to an improvement in the control objective function achieved during the selection process. The algorithm flowchart for our suggested approach to the decision-making process for vehicle control is depicted in Fig. 8.

### Proposed network settings

**Figure 9:**
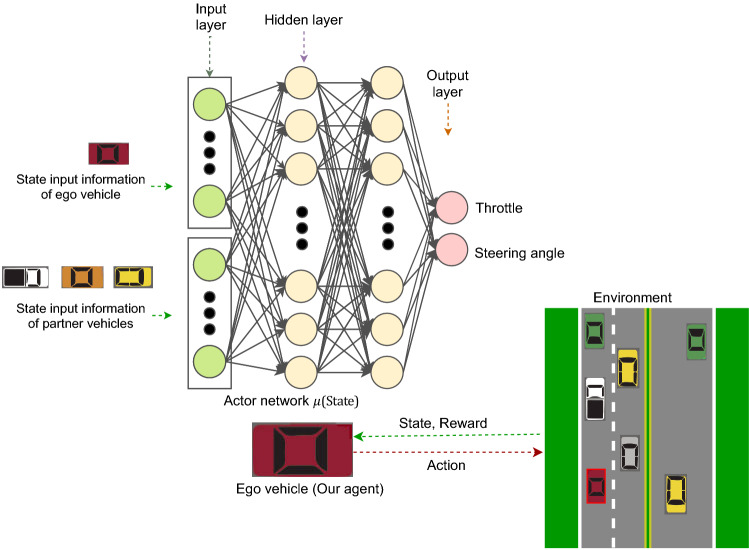
Proposed network settings of the training process.

According to the proposed AI-enable conceptual framework presented in Fig. 7 for MVCCA, the next step is to define the prospective network settings of the suggested training model. For the decision-making and vehicle control processes, this network architecture has two basic elements: a neural network setting and a simulation environment. In particular, in dealing with the neighboring partners in order to achieve a high-level policy for decision-making in MVCCA, three RL algorithms would be used to compare the training performance. The suggested simulator will be Unity3D Game Engine and a multi-agent environment will be created to collaborate with a partner training agent in a high-fidelity traffic environment suite to deploy the simulation. The multiple vehicles in the context of a multi-agent traffic environment that includes various road networks and various traffic task setups will be executed. Figure 9 represents the learning model of the proposed training phase of the system.

In the training period, the ego vehicle first obtains feedback from the environment by way of our control rewards for the safety, smooth, and efficient driving actions of the ego vehicle and the state of its surrounding partner vehicles, and these environments are transmitted through the network. Next, the self-wheel determined the actions longitudinal, and lateral based on a defined policy network and subsequently returned the action to the simulation environment to model the movement and measure the corresponding reward in the next step. The award function integrates the key objectives of the proposed architecture, which are to develop a safe, efficient, and comfort-based automated collision avoidance strategy. In order to achieve the best results, the following factors must be prioritized: (1) to comfort: assessment of jerk (depends on its lateral and longitudinal movement), (2) to efficiency: estimation of total time and distance between participants, and (3) to safety: assessment of collision and near-collision risk.

### Assumption of computational complexity of proposed model

The conceptual framework has a particular proposed training model, and to get a proper assumption about the model complexity or the computational cost of this model, we can follow the prominent model complexity determining notation as $O(\Psi )$,

where $$\Psi =OH+OMY+HI+MYI$$

i.e.

7 $$\begin{aligned} O(\Psi )=O(GH+GMY+HI+MYI) \end{aligned}$$

*G* is the measurement of output units in number, *H* is the total number of used hidden units, *M* is the total number of executed memory cell slabs, *Y* is the magnitude of deployed memory cell slabs, and *I* is the maximum count of implemented forward-connected memory cells.

In regard to the structure that we have proposed for the training model, it is possible to state that, in general, the methods (RL algorithms) are local in both time and space. This indicates that the values of the activation that were obtained through the sequence treating stage are not required to be saved or maintained in any way. In addition, the volume of storage space it needs does not change regardless of the length of the sequence that is fed into it. In fact, the primary focus of the simulations was to find ways to keep multiple autonomous vehicles from colliding in a chain reaction.

So, it would be useful if we tested a number of popular RL methods to see how they fared with our particular challenge. It is well known that this type of algorithm typically learns both a Q-function and a policy simultaneously, so we can employ the off-policy methods in which the actor and critic networks are both subject to “soft updates,” also known as “conservative policy iterations.” The other possibility is to employ an off-policy approach that utilizes off-policy updates in tandem with a stable stochastic actor-critic scheme to provide similar support for our training. Since the policy can learn multiple ways of optimal behavior, we will attempt to use it to prevent chain collisions. Lastly, it would be better for the cost-effectiveness of the model if we looked into any of the on-policy updating methods. In these methods, the agent interacts directly with the environment, learns, and then throws away a batch of experiences after a gradient update.

## Open research issues

In the realm of automated driving, we have identified several critical areas for open challenges. We believe that artificial intelligence will play a key role in overcoming these challenges:

1. The proposed framework states that the combination of diverse sensor data becomes essential for a promising sensing system. It is worth mentioning that important advances in object recognition and detection have been reported^171^. However, the existing systems are intended to calculate 2D or 3D bounding boxes for a few trained object classes. As a result, it is expected that future research will focus on bridging the gap between 2D image data and LiDAR-based 3D data, as well as enhancing identified details to allow more objects to be perceived and tracked in real-time.
2. Real-time needs must be addressed to process massive amounts of data acquired from the vehicle’s sensors and update AI method parameters across higher-speed communication connections^172^. The progress in semiconductor chips for self-driving vehicles and the growth of 5G networks can overcome these limitations.
3. The collision risk evaluation system must fundamentally forecast the vehicle’s gesture throughout a time horizon to include short to medium^173^. As noted by various sources, the key focus of deep learning for AVs is perception and the learning process^174^. However, AI is projected to play a considerable role in local trajectory assessment and planning in the coming years.
4. Since the traffic environment is changing, a vehicle might potentially exceed ordinary road restrictions in an emergency or on an expressway. It is therefore vital to thoroughly investigate how to evaluate the content of this uncertain situation. After all, the multi-modalities (mixed traffic systems) and multiple actors dealing with diverse sub-problems, as described previously in section 2, are extremely challenging, and the optimal solution has yet to be revealed. This paper shows that strategic, tactical, and operational collision prevention problems have deeply interacted with and should be handled in an integrated way.
5. It is challenging to incorporate non-linear vehicle dynamics in real-time in high-speed collision situations. More complex circumstances can also be employed for the future performance of collision avoidance systems, like avoiding unexpected slowdowns and abrupt lane changes. The collision evasion system protects the provision of a broad collision scenario that shows the best research problem for the future.
6. Typically, the trained conventional machine learning model cannot capture all critical traffic scenarios. Enormous research uses various data sets, and the diversity of data sets is frequently not assured during data generation. The training package is somewhat comparable and is rarer during training in rare driving circumstances, where the model will most definitely fail. In order to address this issue, future research should focus on the implementation of reinforcement learning approaches in automated driving.
7. Most driving scenarios are classically believed to be resolvable. The other unsolved solutions are corner cases that need a superhuman driver’s judgement and understanding. Deep learning algorithms’ generalization capacities should be strengthened to tackle corner cases. In learning dangerous scenarios, generalization in a learning model is crucial, partly because the training data for such corner instances are rare. This also means the conception of one-shoot and low-shoot learning systems with fewer examples of instruction.
8. It is worth noting that the ability of AI mechanisms to adapt based on experiences has already been demonstrated to understand the vehicles’ control methods parameters, which is a glimmer of optimism. Therefore, an improved approximation of the underlying precise system model shows future research demands of considerable research.
9. In security-critical schemes, the application of protocols depends on learning-based AI techniques currently being debated, bringing closer relationships between computer intelligence and the functional security sectors. The machine learning package is not covered by existing safety standards, such as ISO26262. Despite the introduction of new data-driven layout methods, there are still questions about the stability, explainability, and classification resilience of neural networks and deep neural networks.
10. Many organizations and companies strive for automated driving to find the most effective way of moving from the tentative experimental phase to the commercial phase. Artificial intelligence and machine learning are common methods, and large amounts of data are needed to research using these methods. However, this is dubious as automobile researchers cannot share their resources because they believe their competitive gain would be diminished. In order to solve this issue, core attention is needed to develop policies that will benefit all automated driving research groups equally and enable them to share their progress easily.

## Conclusion and remarks

This manuscript reviewed the most recent advancements in the research and enrichment of AVs systems toward the avoidance of MVCs. After evaluating the rising issues broadly related to dynamic object detection for collision risk prediction and vehicle control for the avoidance of secondary crashes, this article offered an extensive review of the state-of-the-art CA approaches for collision-free driving strategy in AD. Also, the most relevant single-vehicle and multiple-vehicle environments and the most severe traffic conditions were detailed, along with the necessary avoidance techniques. Regarding these methods, the most prevalent and most recent architectures (for perception, communications, risk prediction, decision-making, and vehicle control), with a particular focus on the various architectures of the subsystems and the scant academic improvements in terms of performance metrics, have proposed AVs systems. Apart from that, to bridge the gaps among contemporary probes, this paper offered an AI-enabled conceptual framework and a decision-making model for MVCCA. Finally, the most pressing unresolved issues in the current MVCCA were listed, outlining a concise strategy for AVs developers to create affordable multi-agent automated driving solutions.

The main concern of this paper is chain-collision avoidance, and potential work reveals that it heavily relies on foolproof perception and a critical risk prediction system. It will require extensive research and development to improve identified details so that more objects can be perceived and tracked in real-time. We studied how covert hidden nodes affect relay connections. Investigating platoon model characteristics, we see that they enhance link operation by diminishing data collisions. The platoon model alleviates the data congestion and delays associated with vehicular networks by managing the communications of multiple vehicles. In a critical driving conditions on an expressway, a vehicle may exceed road restrictions due to the massive changing trend of traffic systems. But this uncertain situation must be thoroughly evaluated. Mixed traffic systems as well as multiple actors dealing with diverse sub-problems are challenging, and the optimal solution is still unknown. However, this paper shows how strategic, tactical, and operational collision prevention problems interact and should be handled together. Non-linear vehicle dynamics are difficult to model in high-speed collision situations. Future research on collision avoidance systems can handle unexpected slowdowns and abrupt lane changes.

The massive engagement of modern AI in diverse domains of AVs encourages us to rethink data-driven algorithms for decision-making and control methods instead of conventional methods. In the classical view, most potential problems that may arise while driving can be solved. It would take a driver with superhuman judgment and comprehension to handle the remaining edge cases, which is why they remain unsolved. To better handle special cases, the generalization capabilities of deep learning algorithms should be improved. Learning potentially harmful scenarios requires a learning model that can generalize well, in part because data for extreme outliers is scarce in training. This entails the development of low-shoot and one-shoot learning systems that provide fewer examples of instruction.

It is hoped that this investigation will provide insight into how to ensure that the different modules of MVCCA cooperate effectively in AVs to achieve the desired driving strategy. Also, researchers and developers will be able to use the article as a reference as they try to improve threat assessment and the way fully automated vehicles are controlled.

## Data Availability

We do not have any data to show in this study.

## Appendix

Table 9 shows the acronyms used in this manuscript for machine learning and automated multiple vehicle collisions related terms.

**Table 9:** Machine learning and automated MVCs related acronyms.

ML related acronyms		Autonomous MVC related acronyms	
ML	Machine learning	AVs	Automated vehicles
A2C	Advantage actor-critic	AD	Automated driving
A3C	Asynchronous A2C	MVCCA	Multiple vehicle cooperation & collision avoidance
MSE	Mean square error	SAE	Society of Automotive Engineers
BC	Behavior cloning	AVAS	Advanced driver assistance systems
DDPG	Deep DPG	HVs	Human driven vehicles
DNN	Deep neural network	PID	Proportional integral derivative
DP	Dynamic programming	MPC	Model predictive control
DPG	Deterministic PG	MAS	Multi-agent systems
DQN	Deep Q-network	CV	Conventional vehicle
DRQN	Deep recurrent Q-network	AE	Auto encoder
HRL	Hierarchical RL	TC	Time to collision
MDP	Markov decision process	SVC	Single vehicle collision
POMDP	Partial MDP	MVCs	Multiple Vehicle Collisions
TD	Temporal difference	CCA	Car accident avoidance
PPO	Proximal policy approximation	CM	Collision mitigation
TRPO	Trust Region Policy Optimization	IMU	Inertial measurement unit
MARL	Multi agent RL	VANET	Vehicular Ad-hoc network
GNNs	Graph neural networks	TAS	Threat assessment systems
MIQP	Mixed-integer quadratic program	CA	Collision avoidance
LSTM	Long-short term memory	LC	Lane change
ReLU	Rectified linear unit	CAV	Connected and automated driving
